# Supplementary material for: pH selects for distinct N2O-reducing microbiomes in tropical soil microcosms
Source: ISME Commun. 2024 May 8;4(1):ycae070. doi: 10.1093/ismeco/ycae070 (PMC11131594; doi:10.1093/ismeco/ycae070)
Supplement: Revised_SI_N2O_31_ycae070 [file revised_si_n2o_31_ycae070.docx]

**Supporting Information**

**pH selects for distinct N_2_O-reducing microbiomes in tropical soil microcosms**

Yanchen Sun^1,2†^, Yongchao Yin^2,3‖^, Guang He^4^, Gyuhyon Cha^5^, Héctor L. Ayala-del-Río^6^, Grizelle González^7^, Konstantinos T. Konstantinidis^5^, and Frank E. Löffler^1,2,3,4^*

^1^Department of Civil and Environmental Engineering, ^2^Center for Environmental Biotechnology, ^3^Department of Microbiology, ^4^Department of Biosystems Engineering and Soil Science, University of Tennessee, Knoxville, Tennessee 37996, USA

^5^School of Civil and Environmental Engineering, Georgia Institute of Technology, Atlanta, Georgia 30332, USA

^6^Department of Biology, University of Puerto Rico at Humacao, Humacao, Puerto Rico, 00792, USA

^7^USDA Forest Service, International Institute of Tropical Forestry, Río Piedras, Puerto Rico, 00926, USA

^†^Current address: Department of Marine Chemistry and Geochemistry, Woods Hole Oceanographic Institution, Woods Hole, Massachusetts 02543, USA

^‖^Current address: Department of Biology, Antimicrobial Discovery Center, Northeastern University, Boston, Massachusetts 02148, USA

***Corresponding author**: Frank E. Löffler, University of Tennessee, Department of Civil and Environmental Engineering, 325 John D. Tickle Building, 851 Neyland Drive, Knoxville, TN 37996, USA. Phone: (865) 974-4933, E-mail: [frank.loeffler@utk.edu](mailto:frank.loeffler@utk.edu)

**Supporting Information summary:** 10 tables, 12 figures, and references.

**Table S1.** Physicochemical properties of soil samples collected along an elevational gradient in the Luquillo Experimental Forest (LEF) in Puerto Rico [1].

| Sampling location | Abbreviation | Moisture | Class | pH | Total C | Total N |  | NO_3_-N | NO_2_-N | NH_4_-N | Sulfate |
| --- | --- | --- | --- | --- | --- | --- | --- | --- | --- | --- | --- |
|  |  |  |  |  | % *^a^* | |  | mg kg^-1^ *^b^* | | | |
| Sabana | S | 30.70% | Clay | 4.72 | 1.717 | 0.167 |  | < 0.190 | < 0.210 | 2.39 | 3.43 |
| El Verde | EV | 33.80% | Clay | 4.45 | 3.698 | 0.246 |  | < 0.190 | < 0.210 | 3.78 | 2.31 |
| Palm Nido | PN | 48.90% | Clay Loam | 4.97 | 6.604 | 0.280 |  | < 0.190 | < 0.210 | 5.52 | 10.08 |
| Pico del Este | PE | 54.20% | Silty Clay Loam | 4.67 | 8.489 | 0.338 |  | < 0.190 | < 0.210 | 1.16 | 6.46 |

*^a^* Reported as percent of dry weight soil.

*^b^* Reported in mg kg^-1^ (i.e., wet weight of soil at the time of sampling).

S, Sabana (265 m above mean sea level [MSL]); EV, El Verde (453 m MSL); PN, Palm Nido (634 m MSL); PN, Pico del Este (953 m MSL).

Additional information about of the vegetation and soil characteristics of the LEF sampling locations are available in the published literature [2, 3].

**Table S2.** Information about the metagenome data generated from the 16 N_2_O-reducing tropical soil microcosms.

|  |  |  | **Location** | | | |
| --- | --- | --- | --- | --- | --- | --- |
|  |  |  | **S** | **EV** | **PN** | **PE** |
| Low level N_2_O | pH 4.5 | NCBI SRA No. | SRR22334937 | SRR22334946 | SRR22334945 | SRR22334938 |
|  |  | Number of reads | 52 255 324 | 63 252 210 | 61 708 064 | 69 517 460 |
|  | pH 7.3 | NCBI SRA No. | SRR22334943 | SRR22334932 | SRR22334933 | SRR22334944 |
|  |  | Number of reads | 56 398 178 | 61 230 092 | 62 455 532 | 57 257 032 |
| High level N_2_O | pH 4.5 | NCBI SRA No. | SRR22334933 | SRR22334936 | SRR22334935 | SRR22334934 |
|  |  | Number of reads | 58 035 950 | 54 289 774 | 72 962 738 | 57 417 756 |
|  | pH 7.3 | NCBI SRA No. | SRR22334939 | SRR22334942 | SRR22334941 | SRR22334940 |
|  |  | Number of reads | 60 836 792 | 47 671 962 | 57 093 952 | 58 052 954 |

**Table S3.** Metadata for published metagenomes downloaded from the European Nucleotide Archive.

| **Biome** | **Location** | **pH** | **NCBI SRA No.** | **Number of samples** | **Reference** |
| --- | --- | --- | --- | --- | --- |
| Temperate forest | British Columbia, Canada | 5.0-5.5 | ERR753925  ERR753926  ERR753927 | 3 | [4] |
| Tropical forest | Gigante Peninsula, Panama | 4.5 | SRR5262244  SRR5262250 | 2 | [5] |
| Temperate forest | New Hampshire, United States | 4.5 | SRR5580658  SRR5580692 | 2 | [6] |
| Tropical forest | Mount Kilimanjaro, Tanzania | 4.9-5.2 | ERR4660996  ERR4660997  ERR4660998  ERR4660999 | 4 | [7] |
| Agricultural land | Yunnan, China | 3.5-5.2 | SRR10260009  SRR10260010 | 2 | [8] |
| Temperate forest | Indiana, USA | 5.7 | SRR8437983  SRR7687007  SRR8437984  SRR7687014 | 4 | [9] |
| Permafrost | Canada | 5.5 | SRR1586253  SRR1586257  SRR1586264 | 3 | [10] |
| Agricultural land | Norway | 3.8-4.0 | ERR5023167  ERR5023168  ERR5023169 | 3 | [11] |
| *^a^*Tropical forest | Puerto Rico | 4.5 | Not available | 4 | [1] |
| Agricultural land | Illinois, USA | 6.1-7.5 | ERR1939172  ERR1939173  ERR1939174  ERR1939267  ERR1939269 | 5 | [12] |
| Tropical forest | Mount Kilimanjaro (RAU), Tanzania | 7.5 | ERR4661012  ERR4661013  ERR4661014 | 3 | [7] |

*^a^* The metagenomic datasets were deposited in the European Nucleotide Archive (ENA) under project PRJEB26500.

**Table S4.** Performance of microcosms established with tropical soils collected along an elevational gradient in the LEF and experiencing low versus high N_2_O concentrations and acidic versus circumneutral pH conditions.

|  |  |  | EV | PN | PE | S |
| --- | --- | --- | --- | --- | --- | --- |
|  |  | N_2_O |  |  |  |  |
| Low level N_2_O | pH 4.5 | # of feedings | 26 | 18 | 21 | 21 |
|  |  | Total amount (µmol) | 108 | 75 | 87 | 87 |
|  | pH 7.3 | # of feedings | 34 | 26 | 29 | 31 |
|  |  | Total amount (µmol) | 141 | 108 | 120 | 129 |
| High level N_2_O | pH 4.5 | # of feedings | 8 | 8 | 5 | 7 |
|  |  | Total amount (µmol) | 3 328 | 3 328 | 2 080 | 2 912 |
|  | pH 7.3 | # of feedings | 17 | 15 | 9 | 13 |
|  |  | Total amount (µmol) | 7 072 | 6 240 | 3 744 | 5 408 |

**Table S5.** Number of 16S rRNA gene sequences derived from the original soils and 16 N_2_O-reducing microcosms.

|  | **EV** | **PN** | **PE** | **S** |
| --- | --- | --- | --- | --- |
| Original soil | 4 390 | 7 483 | 8 636 | 3 341 |
| pH4.5_0.1 | 18 442 | 11 514 | 13 988 | 59 035 |
| pH4.5_10 | 23 100 | 14 905 | 19 532 | 55 570 |
| pH7.3_0.1 | 12 235 | 16 713 | 12 631 | 23 729 |
| pH7.3_10 | 9 792 | 20 144 | 23 505 | 39 554 |

The numbers 0.1 and 10 reflect low (0.02 mM) and high (2 mM) N_2_O levels in the microcosms.

**Table S6.** Results of PERMANOVA based on weighted-UniFrac distance for the effects of pH and N_2_O on microbial community composition.

| **Factor** | **Df** | **Sum of Squares** | **R^2^** | **F** | ***p*-value** |
| --- | --- | --- | --- | --- | --- |
| pH | 2 | 1.083 | 0.375 | 4.965 | 0.001 |
| N_2_O | 1 | 0.108 | 0.037 | 0.986 | 0.406 |
| pH × N_2_O | 1 | 0.059 | 0.020 | 0.539 | 0.861 |

**Table S7.** Number of *nosZ* reads identified in metagenome datasets derived from N_2_O-reducing tropical soil microcosms using ROCker models.

|  |  |  | | **Number of *nosZ*** **Sequences** | | | | | | | |
| --- | --- | --- | --- | --- | --- | --- | --- | --- | --- | --- | --- |
|  |  | **Clade I** | | | | |  | **Clade II** | | | |
|  |  | **Location** | | | | |  | **Location** | | | |
| **Microcosm** |  | **S** | **EV** | | **PN** | **PE** |  | **S** | **EV** | **PN** | **PE** |
| pH4.5_0.1 |  | 1 065 | 123 | | 4 151 | 3 283 |  | 1 626 | 720 | 5 833 | 7 585 |
| pH4.5_10 |  | 8 | 105 | | 16 506 | 245 |  | 69 | 3 479 | 22 940 | 3 529 |
| pH7.3_0.1 |  | 1 574 | 147 | | 174 | 252 |  | 312 | 1 487 | 5 436 | 2 443 |
| pH7.3_10 |  | 6 140 | 58 | | 2 913 | 171 |  | 31 604 | 474 | 10 420 | 7 165 |

The numbers 0.1 and 10 reflect low (0.02 mM) and high (2 mM) N_2_O levels in the microcosms.

**Table S8.** Summary statistics of the 17 high-quality MAGs derived from N_2_O-reducing microcosms harboring *nosZ* genes.

| **MAG ID *^a^*** | **Genome size (Mbp)** | **No. scaffolds** | **N50 (scaffolds)** | **GC (%)** | **No. CDS *^b^*** | **Completeness (%)** | **Contamination (%)** |
| --- | --- | --- | --- | --- | --- | --- | --- |
| PN_pH4.5_0.1_MAG1 | 6.72 | 547 | 22792 | 68.6 | 6344 | 97.05 | 1.74 |
| PE_pH4.5_0.1_MAG2 | 6.39 | 623 | 15910 | 68.4 | 6109 | 90.22 | 3.01 |
| S_pH4.5_0.1_MAG3 | 6.31 | 1038 | 8619 | 68.1 | 6441 | 84.89 | 3.85 |
| EV_pH4.5_10_MAG4 | 5.42 | 139 | 97917 | 46.3 | 5146 | 99.43 | 2.69 |
| PN_pH4.5_10_MAG5 | 5.34 | 126 | 139753 | 46.4 | 5057 | 99.43 | 1.62 |
| PE_pH4.5_10_MAG6 | 4.62 | 107 | 98082 | 42.9 | 4372 | 100 | 1.28 |
| PE_pH4.5_10_MAG7 | 7.67 | 253 | 73205 | 51.6 | 6926 | 98.39 | 2.26 |
| PE_pH4.5_10_MAG8 | 2.55 | 570 | 6301 | 68.2 | 2774 | 84.03 | 2.36 |
| EV_pH7.3_0.1_MAG9 | 3.62 | 40 | 224397 | 63.8 | 3314 | 99.76 | 0.32 |
| PN_pH7.3_0.1_MAG10 | 3.69 | 42 | 405271 | 63.3 | 3363 | 99.76 | 0.95 |
| PE_pH7.3_0.1_MAG11 | 3.90 | 108 | 339534 | 63.1 | 3605 | 99.76 | 1.76 |
| S_pH7.3_0.1_MAG12 | 3.19 | 70 | 151307 | 62.5 | 3080 | 98.66 | 0.71 |
| EV_pH7.3_10_MAG13 | 5.34 | 183 | 70882 | 46.2 | 5090 | 84.18 | 2.17 |
| PN_pH7.3_10_MAG14 | 3.55 | 71 | 122344 | 63.5 | 3404 | 100 | 0.05 |
| PE_pH7.3_10_MAG15 | 3.45 | 44 | 115048 | 63.9 | 3281 | 98.34 | 0.43 |
| S_pH7.3_10_MAG16 | 3.58 | 82 | 72588 | 63.8 | 3321 | 98.48 | 0.32 |
| S_pH7.3_10_MAG17 | 6.80 | 86 | 206838 | 47.5 | 5053 | 98.89 | 3.13 |

***^a^*** The numbers 0.1 and 10 reflect low (0.02 mM) and high (2 mM) N_2_O levels in the microcosms.

***^b^*** CDS, coding sequence, is a region of DNA whose sequence codes for a protein.

**Table S9.** GTDB-Tk taxonomic classification of the taxa represented by the 17 high-quality MAGs harboring *nosZ* genes.

| **MAG ID *^a^*** | **GTDB-Tk taxonomic classification** |
| --- | --- |
| PN_pH4.5_0.1_MAG1 | d__Bacteria;p__Proteobacteria;c__Alphaproteobacteria;o__Rhizobiales;f__Xanthobacteraceae;g__Rhodoplanes;s__ |
| PE_pH4.5_0.1_MAG2 | d__Bacteria;p__Proteobacteria;c__Alphaproteobacteria;o__Rhizobiales;f__Xanthobacteraceae;g__Rhodoplanes;s__ |
| S_pH4.5_0.1_MAG3 | d__Bacteria;p__Proteobacteria;c__Alphaproteobacteria;o__Rhizobiales;f__Xanthobacteraceae;g__Rhodoplanes;s__ |
| EV_pH4.5_10_MAG4 | d__Bacteria;p__Firmicutes_B;c__Desulfitobacteriia;o__Desulfitobacteriales;f__Desulfitobacteriaceae;g__Desulfosporosinus;s__ |
| PN_pH4.5_10_MAG5 | d__Bacteria;p__Firmicutes_B;c__Desulfitobacteriia;o__Desulfitobacteriales;f__Desulfitobacteriaceae;g__Desulfosporosinus;s__ |
| PE_pH4.5_10_MAG6 | d__Bacteria;p__Firmicutes_B;c__Desulfitobacteriia;o__Desulfitobacteriales;f__Desulfitobacteriaceae;g__Desulfosporosinus;s__ |
| PE_pH4.5_10_MAG7 | d__Bacteria;p__Desulfobacterota;c__Desulfomonilia;o__Desulfomonilales;f__Desulfomonilaceae;g__Desulfomonile;s__ |
| PE_pH4.5_10_MAG8 | d__Bacteria;p__Actinobacteriota;c__Coriobacteriia;o__OPB41;f__PALSA-660;g__;s__ |
| EV_pH7.3_0.1_MAG9 | d__Bacteria;p__Proteobacteria;c__Gammaproteobacteria;o__Burkholderiales;f__Rhodocyclaceae;g__Azospira;s__ |
| PN_pH7.3_0.1_MAG10 | d__Bacteria;p__Proteobacteria;c__Gammaproteobacteria;o__Burkholderiales;f__Rhodocyclaceae;g__Azospira;s__ |
| PE_pH7.3_0.1_MAG11 | d__Bacteria;p__Proteobacteria;c__Gammaproteobacteria;o__Burkholderiales;f__Rhodocyclaceae;g__Azospira;s__ |
| S_pH7.3_0.1_MAG12 | d__Bacteria;p__Proteobacteria;c__Gammaproteobacteria;o__Burkholderiales;f__SulfuricEVlaceae;g__UBA2239;s__ |
| EV_pH7.3_10_MAG13 | d__Bacteria;p__Firmicutes_B;c__Desulfitobacteriia;o__Desulfitobacteriales;f__Desulfitobacteriaceae;g__Desulfosporosinus;s__ |
| PN_pH7.3_10_MAG14 | d__Bacteria;p__Proteobacteria;c__Gammaproteobacteria;o__Burkholderiales;f__Rhodocyclaceae;g__;s__ |
| PE_pH7.3_10_MAG15 | d__Bacteria;p__Proteobacteria;c__Gammaproteobacteria;o__Burkholderiales;f__Rhodocyclaceae;g__Azospira;s__ |
| S_pH7.3_10_MAG16 | d__Bacteria;p__Proteobacteria;c__Gammaproteobacteria;o__Burkholderiales;f__Rhodocyclaceae;g__Azospira;s__ |
| S_pH7.3_10_MAG17 | d__Bacteria;p__Firmicutes_B;c__Desulfitobacteriia;o__Desulfitobacteriales;f__Desulfitobacteriaceae;g__Desulfitobacterium;s__ |

***^a^*** The numbers 0.1 and 10 reflect low (0.02 mM) and high (2 mM) N_2_O levels in the microcosms.

**Table S10.** Analysis of 17 high-quality MAGs harboring *nosZ* genes using the Microbial Genome Atlas (MiGA). Listed for each MAG is the average amino acid identity (AAI), the closest relative, and the novelty of each MAG. The taxonomic novelty is determined by the maximum AAI value compared to genomes in the TypeMat database. The p-value is estimated from the empirical distribution observed in all reference genomes in the NCBI Reference Sequence Database (RefSeq) at each taxonomic level and indicates the probability of the observed AAI between genomes of the same taxon. The TypeMat database contains assemblies from type materials in Archaea and Bacteria (as flagged by NCBI) including both complete and draft genomes.

| **MAG ID *^a^*** | **Best classification level** | **AAI** | **Closest relative** | **Novelty** |
| --- | --- | --- | --- | --- |
| PN_pH4.5_0.1_MAG1 | **Order** *Rhizobiales* (p-value: 0.0024) | 71.28 | *Rhodoplanes roseus* | **Species** (p-value: 0.00252) |
| PE_pH4.5_0.1_MAG2 | **Order** *Rhizobiales* (p-value: 0.0024) | 71.17 | *Rhodoplanes roseus* | **Species** (p-value: 0.00252) |
| S_pH4.5_0.1_MAG3 | **Order** *Rhizobiales* (p-value: 0.0024) | 70.32 | *Rhodoplanes roseus* | **Species** (p-value: 0.00252) |
| EV_pH4.5_10_MAG4 | **Family** *Peptococcaceae* (p-value: 0.0057) | 78.82 | *Desulfosporosinus orientis* DSM 765 | **Species** (p-value: 0.00269) |
| PN_pH4.5_10_MAG5 | **Family** *Peptococcaceae* (p-value: 0.0057) | 79.2 | *Desulfosporosinus orientis* DSM 765 | **Species** (p-value: 0.00269) |
| PE_pH4.5_10_MAG6 | **Family** *Peptococcaceae* (p-value: 0.0061) | 77.84 | *Desulfosporosinus lacus* DSM 15449 | **Species** (p-value: 0.00257) |
| PE_pH4.5_10_MAG7 | **Phylum** Proteobacteria (p-value: 0.0011) | 45.04 | *Desulfosoma caldarium* | **Family** (p-value: 0.0151) |
| PE_pH4.5_10_MAG8 | **Phylum** Actinobacteria (p-value: 0.0011) | 44.04 | *Rhabdothermincola sediminis* | **Family** (p-value: 0.0119) |
| EV_pH7.3_0.1_MAG9 | **Order** *Rhodocyclales* (p-value: 0.0024) | 69.99 | *Azospira oryzae* | **Species** (p-value: 0.00252) |
| PN_pH7.3_0.1_MAG10 | **Order** *Rhodocyclales* (p-value: 0.0024) | 69.98 | *Azospira oryzae* | **Species** (p-value: 0.00252) |
| PE_pH7.3_0.1_MAG11 | **Order** *Rhodocyclales* (p-value: 0.0024) | 69.99 | *Azospira oryzae* | **Species** (p-value: 0.00252) |
| S_pH7.3_0.1_MAG12 | **Class** *Betaproteobacteria* (p-value: 0.00073) | 56.82 | *Sulfurimicrobium lacus* NZ AP022853 | **Species** (p-value: 0.000457) |
| EV_pH7.3_10_MAG13 | **Family** *Peptococcaceae* (p-value: 0.0057) | 79.42 | *Sulfurimicrobium lacus* NZ AP022853 | **Species** (p-value: 0.00269) |
| PN_pH7.3_10_MAG14 | **Order** *Rhodocyclales* (p-value: 0.0021) | 66.86 | *Dechloromonas hortensis* | **Species** (p-value: 0.002) |
| PE_pH7.3_10_MAG15 | **Order** *Rhodocyclales* (p-value: 0.0024) | 69.21 | *Azospira oryzae* | **Species** (p-value: 0.00252) |
| S_pH7.3_10_MAG16 | **Order** *Rhodocyclales* (p-value: 0.0024) | 69.64 | *Azospira oryzae* | **Species** (p-value: 0.00252) |
| S_pH7.3_10_MAG17 | **Genus** *Desulfitobacterium* (p-value: 0.0079) | 89.81 | *Desulfitobacterium chlororespirans* DSM 11544 | **Subspecies** (p-value: 0.000182) |

***^a^*** The numbers 0.1 and 10 reflect low (0.02 mM) and high (2 mM) N_2_O levels in the microcosms.

**Figure S1.** Schematic of the study site and overview of the experimental design. The numbers 0.1 and 10 represent the low and high levels of N_2_O maintained in the microcosms, respectively. Microcosms had two technical replicates, which showed similar performance, and one microcosm per treatment was selected for metagenome sequencing (16 total). Negative controls included heat-killed (autoclaved) replicates and microcosms without N_2_O but with lactate at pH 4.5 for each soil sample. The metagenomes of the four original soils have been analyzed in a prior study [1] and are available in the European Nucleotide Archive under project PRJEB26500.

**Figure S2.** Phylogenetic diversity of the putative near full-length *nosZ* sequences recovered from the assembled contigs and *nosZ* from the reference *nosZ* database. The red half circle indicates clade I *nosZ* and the green half circle depicts clade II *nosZ*. Branch labels in blue represent the *nosZ* contigs assembled from the metagenomic data. The numbers 0.1 and 10 reflect low (0.02 mM) and high (2 mM) N_2_O levels in the microcosms.

**Figure S3.** Phylogenetic diversity of the near full-length *nosZ* sequences recovered from the high-quality MAGs and *nosZ* from the reference *nosZ* database. The red and the green half circles indicate clade I *nosZ* and clade II *nosZ*, respectively. Branch labels in blue indicate *nosZ* sequences from the MAGs. The numbers 0.1 and 10 reflect low (0.02 mM) and high (2 mM) N_2_O levels in the microcosms.

**Figure S4.** N_2_O reduction profiles in acidic (pH 4.5) tropical soil microcosms. The numbers 0.1 and 10 reflect low (0.02 mM) and high (2 mM) N_2_O levels in the microcosms. Replicate microcosms showed similar performance.

**Figure S5.** Rarefaction curves of 16S rRNA gene sequences recovered from metagenomic datasets. Curves represent the number of unique OTUs recovered, defined at the 97% nucleotide sequence identity level, for the number of sequences analyzed, and reflect the extent of OTU diversity within the samples and what fraction of this diversity was sampled.

**Figure S6.** Microbial community compositions based on 16S rRNA gene fragments recovered from metagenome data from original LEF soils and the N_2_O-reducing microcosms maintained at pH 4.5 and pH 7.3 and with low (0.02 mM) and high (2 mM) levels of N_2_O. (A) Relative abundances of the top 20 families in the original soils and the microcosms maintained under the different enrichment conditions. (B) Relative abundances of the top 20 genera in the original soils and the microcosms maintained under the different enrichment conditions. The numbers 0.1 and 10 reflect low (0.02 mM) and high (2 mM) N_2_O levels in the microcosms.

**Figure S7.** Abundance of *nosZ* genes in the original LEF soils and the 16 N_2_O-reducing microcosms selected for metagenome sequencing. The numbers 0.1 and 10 reflect low (0.02 mM) and high (2 mM) N_2_O levels in the microcosms.

**Figure S8.** Differences in microbial community composition between the original soils and the respective N_2_O-reducing microcosms based on weighted Unifrac analysis of *nosZ* gene fragments recovered in the metagenomes using ROCker. (A) Beta diversity based on clade II *nosZ* sequences. (B) Beta diversity based on clade I *nosZ* sequences N_2_O. Samples are visualized by principal coordinate analysis (PCoA) with colors representing the original soil (blue) and pH (pH 4.5, red; pH 7.3, green). The ellipses represent the 95% confidence intervals.

**Figure S9.** The relative abundance of each MAG harboring a *nosZ* gene derived from N_2_O-reducing microcosms in the corresponding original soils based on metagenomic reads competitively mapped against the MAGs.

**Figure S10.** The relative abundance of MAGs harboring a *nosZ* gene derived from acidic microcosms and detected in the corresponding neutral microcosm (left panel), and MAGs harboring a *nosZ* gene derived from neutral microcosms and detected in the corresponding acidic microcosm (right panel).

**Figure S11.** Comparison of *nos* clusters in the 17 MAGs harboring *nosZ* genes derived from N_2_O-reducing microcosms. *nos* cluster genes were annotated using the SEED subsystem and validated by using the BLAST. Transmembrane helices in transmembrane proteins were identified using the TMHMM2 (<http://smart.embl-heidelberg.de/>). cy-b and cy-c represent b-type and c-type cytochromes, respectively. Fe-S, S, and TM represent genes encoding iron-sulfur-binding proteins, Rieske iron-sulfur proteins, and transmembrane proteins, respectively. The numbers 0.1 and 10 reflect low (0.02 mM) and high (2 mM) N_2_O levels in the microcosms. The numbers 1 and 2 after the last low dash in the left label represent the first and second *nosZ* genes identified in the corresponding MAG.

**Figure S12.** Relationships between pH and the total abundance of *nosZ* genes per genome equivalent. Detailed information about the metagenome datasets is provided in Table S3. *nosZ* genes were searched against a customized database of *nosZ* based on near full-length *nosZ* identified in the acidic N_2_O-reducing microcosms. The plots show the total abundance of *nosZ* genes per genome equivalent. Gray areas represent 95% confidence intervals.

**References**

1. Karthikeyan S, Orellana LH, Johnston ER, Hatt JK, Löffler FE, Ayala-del-Río HL, et al. Metagenomic characterization of soil microbial communities in the Luquillo experimental forest (Puerto Rico) and implications for nitrogen cycling. Appl Environ Microbiol. 2021;87:e00546-00521.

2. Gould W, González G, Carrero Rivera G. Structure and composition of vegetation along an elevational gradient in Puerto Rico. J Veg Sci. 2006;17:653-664.

3. Ping C-L, Michaelson GJ, Stiles CA, González G. Soil characteristics, carbon stores, and nutrient distribution in eight forest types along an elevation gradient, eastern Puerto Rico. Ecol Bull. 2013:67-86.

4. Cardenas E, Kranabetter J, Hope G, Maas KR, Hallam S, Mohn WW. Forest harvesting reduces the soil metagenomic potential for biomass decomposition. ISME J. 2015;9:2465-2476.

5. Yao Q, Li Z, Song Y, Wright SJ, Guo X, Tringe SG, et al. Community proteogenomics reveals the systemic impact of phosphorus availability on microbial functions in tropical soil. Nat Ecol Evol. 2018;2:499-509.

6. Garcia MO, Templer PH, Sorensen PO, Sanders-DeMott R, Groffman PM, Bhatnagar JM. Soil microbes trade-off biogeochemical cycling for stress tolerance traits in response to year-round climate change. Front Microbiol. 2020;11:616.

7. Dai Z, Zang H, Chen J, Fu Y, Wang X, Liu H, et al. Metagenomic insights into soil microbial communities involved in carbon cycling along an elevation climosequences. Environ Microbiol. 2021;23:4631-4645.

8. Li H, Wang H, Tao X, Wang X, Jin W, Gilbert JA, et al. Continental-scale paddy soil bacterial community structure, function, and biotic interaction. mSystems. 2021;6:e01368-01320.

9. Kelly CN, Schwaner GW, Cumming JR, Driscoll TP. Metagenomic reconstruction of nitrogen and carbon cycling pathways in forest soil: Influence of different hardwood tree species. Soil Biol Biochem. 2021;156:108226.

10. Wu X, Chauhan A, Layton AC, Lau Vetter MC, Stackhouse BT, Williams DE, et al. Comparative metagenomics of the active layer and permafrost from low-carbon soil in the canadian high arctic. Environ Sci Technol. 2021;55:12683-12693.

11. Frostegård Å, Vick SH, Lim NY, Bakken LR, Shapleigh JP. Linking meta-omics to the kinetics of denitrification intermediates reveals pH-dependent causes of N_2_O emissions and nitrite accumulation in soil. ISME J. 2022;16:26-37.

12. Orellana LH, Chee-Sanford JC, Sanford RA, Löffler FE, Konstantinidis KT. Year-round shotgun metagenomes reveal stable microbial communities in agricultural soils and novel ammonia oxidizers responding to fertilization. Appl Environ Microbiol. 2018;84:e01646-01617.
